# Review article: Immunosuppression in Decompensated Autoimmune Hepatitis Cirrhosis ‐ To Suppress or Not to Suppress?

**DOI:** 10.1111/apt.70893

**Published:** 2026-08-03

**Authors:** Pedro Robson Costa Passos, Valbert Oliveira Costa Filho, Guilherme Grossi Lopes Cançado

**Affiliations:** ^1^ Center of Research and Drug Development (NPDM), Federal University of Ceará Fortaleza Ceará Brazil; ^2^ Biomedical Research Center (NUBIMED), Federal University of Ceará Fortaleza Ceará Brazil; ^3^ Hospital das Clínicas Federal University of Minas Gerais Belo Horizonte Minas Gerais Brazil; ^4^ Hospital da Polícia Militar de Minas Gerais Belo Horizonte Minas Gerais Brazil

**Keywords:** autoimmune hepatitis, cirrhosis, immunosuppression, liver decompensation, recompensation

## Abstract

**Background:**

The management of decompensated autoimmune hepatitis (AIH) cirrhosis is one of the most challenging and controversial scenarios in hepatology. Clinicians face a high‐stakes dilemma: whether to initiate immunosuppression to halt inflammatory activity or prioritize liver transplantation (LT) referral to avoid treatment futility and life‐threatening infections.

**Aims:**

To critically appraise the evidence regarding immunosuppression in decompensated AIH cirrhosis and propose a biologically grounded, risk‐stratified framework for clinical decision‐making.

**Methods:**

This review synthesizes current observational data and retrospective studies, focusing on patient selection, the ‘therapeutic window’ for medical intervention and the physiological impact of cirrhosis‐associated immune dysfunction.

**Results:**

The risks of infection, treatment futility and delayed transplant access must be weighed against the possibility of halting inflammatory activity and achieving clinical stabilization. A narrow therapeutic window exists for medical therapy. Clinical ‘recompensation’ is achievable in 19%–31% of cases, though this is less frequent than in other etiologies. Early dynamic response assessment—specifically changes in bilirubin and MELD‐Na at weeks 1 and 4—helps distinguish responders from those requiring urgent LT. While LT offers excellent long‐term survival, recurrence occurs in 20% of cases.

**Conclusions:**

Management must shift from a ‘one‐size‐fits‐all’ approach to an individualized, protocolized strategy. We advocate for a time‐limited trial of immunosuppression in selected candidates, governed by strict ‘stop rules’ and immediate LT evaluation for non‐responders or high‐risk patients.

## Graphical abstract

Individualized assessment identifies patients with decompensated autoimmune hepatitis cirrhosis who are likely to benefit from a time‐limited trial of immunosuppression versus those requiring immediate liver transplant evaluation. Early objective response monitoring (bilirubin, MELD‐Na and AST/ALT) guides continuation of immunosuppression, promotes recompensation in responders and minimizes delays to life‐saving liver transplantation in non‐responders.

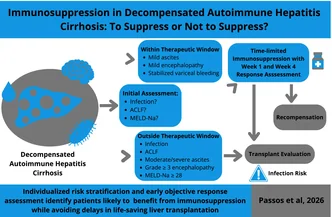

**Abbreviations**
ACLF: acute‐on‐chronic liver failure
AIH: autoimmune hepatitis
ALT: alanine aminotransferase
AS‐AIH: acute severe autoimmune hepatitis
AST: aspartate aminotransferase
CAID: cirrhosis‐associated immune dysfunction
CBR: complete biochemical remission
CD: cluster of differentiation
DAMP: damage‐associated molecular pattern
GGT: gamma‐glutamyl transferase
Hb: haemoglobin
HCC: hepatocellular carcinoma
HLA: human leukocyte antigen
HR: hazard ratio
IAIHG: International Autoimmune Hepatitis Group
IFN‐γ: interferon gamma
IgG: immunoglobulin G
IL: interleukin
INR: international normalized ratio
LPS: lipopolysaccharide
LSEC: liver sinusoidal endothelial cell
LT: liver transplantation
MAdCAM‐1: mucosal addressin cell adhesion molecule 1
MELD: Model for End‐Stage Liver Disease
MELD‐Na: Model for End‐Stage Liver Disease‐Sodium
mHAI: modified hepatitis activity index
NK: natural killer
OHE: overt hepatic encephalopathy
RNA: ribonucleic acid
SURFASA: Survival and Prognostic Factors for Severe Autoimmune Hepatitis
TFS: transplant‐free survival
Th1: T helper 1
TNF: tumour necrosis factor
Treg: regulatory T cell

## 1 Introduction

Autoimmune hepatitis (AIH) is a rare chronic liver disease that predominantly affects women and is notable for its variable relapsing clinical course [1]. AIH is considered a classical autoimmune liver disease, with inflammation that can range from mild to submassive necrosis and, in severe cases, subacute or acute liver failure [2, 3]. Although recent data suggest that the proportion of patients with cirrhosis at initial presentation has declined from 33% to approximately 10% [4, 5, 6], up to 42% of these patients will experience fibrosis progression at follow‐up [7, 8]. Notably, patients with AIH‐related cirrhosis seem to develop less decompensating events than cirrhosis by other etiologies, especially those who achieve complete biochemical remission (CBR), and may also exhibit better prognosis after decompensation [9, 10].

Despite this, the management of decompensated AIH cirrhosis remains one of the most challenging and controversial areas in the field of liver autoimmune disease therapeutics. The central clinical dilemma is whether immunosuppressive therapy should be pursued while patients await liver transplantation (LT). On one hand, these patients are highly susceptible to infections due to cirrhosis‐associated immune dysfunction (CAID), increased bacterial translocation and portal hypertension‐related complications, risks that may be further amplified by immunosuppressive therapy [11, 12]. On the other hand, effective immunosuppression may attenuate inflammatory activity, stabilize liver function, and, paradoxically, delay or complicate access to LT by reducing Model for End‐Stage Liver Disease (MELD) scores without necessarily restoring true hepatic reserve [13, 14].

Consequently, rapid referral and early listing for LT are generally recommended in cases of severely decompensated AIH, particularly in patients who fail to respond to medical therapy or who present with contraindications to immunosuppression [13, 15]. Transplantation offers excellent long‐term outcomes and remains the definitive treatment for end‐stage AIH [16, 17, 18, 19]. However, the recent introduction of the Baveno VII concept of recompensation has reframed this paradigm. Under specific conditions, namely, effective etiological control and sustained clinical stability, some patients with previously decompensated cirrhosis may revert to a compensated state [20]. Accumulating evidence suggests that patients with decompensated AIH can similarly achieve recompensation following successful immunosuppressive therapy, particularly when treatment is initiated before irreversible organ dysfunction develops [21]. Nevertheless, the available evidence remains limited, heterogeneous and almost exclusively derived from retrospective observational studies, leaving the patients most likely to benefit and the optimal timing of therapy incompletely defined. An individualized decision‐making process on whether to immunosuppress or not, based on the type and severity of decompensation coupled with a prompt evaluation for LT, is currently recommended. In this review, we aim to critically review the literature on immunosuppression in decompensated AIH cirrhosis patients, with a focus on the evolving balance between medical therapy and timely LT.

## 2 Immunological Entanglement

AIH is thought to be a T‐cell disease, mainly caused by a loss of tolerance toward self‐antigens related to regulatory T‐cell (Treg) dysfunction, as well as clonally expanded liver‐resident CD4+ and CD8+ T‐cell populations [22, 23]. Under these conditions, Tregs are primarily defective in function. Although some studies have reported reduced Treg numbers, particularly at diagnosis and during relapses, these findings remain inconsistent, and functional impairment is considered the hallmark of Treg dysregulation in AIH [24]. This allows for expansion of effector subsets, particularly Th1 cells [25]. These cells secrete IFN‐γ and IL‐2, which induce increased HLA class I and aberrant HLA class II expression on hepatocytes [26, 27, 28, 29]. Moreover, increased IFN‐γ activity stimulates CD8+ cytotoxic T cells, which directly mediate hepatocyte destruction [30]. In addition, tumour necrosis factor (TNF), the most upregulated cytokine in AIH liver specimens compared with controls, mediates tissue injury and apoptosis by amplifying macrophage and natural killer (NK) T‐cell functions that are resistant to Treg inhibition [2, 31]. TNF‐producing Th1 cells are selectively expanded in liver infiltrates of AIH patients [32]. Recent single‐cell transcriptomic, spatial transcriptomic, and functional analyses have refined this paradigm by identifying clonally expanded tissue‐resident type 1 memory CD4+ T cells as a major intrahepatic source of TNF and IFN‐γ. These cells interact with auto‐aggressive CD8+ T cells, hepatocytes and myeloid cells through TNF‐dependent signalling [23].

As fibrosis progresses toward cirrhosis, this Th1‐mediated inflammatory milieu is amplified by a parallel systemic trigger. Translocation of enteric bacterial products, leading to increased IFN‐γ production by Th1‐polarized cells, is one of the greatest lines of evidence for systemic inflammation in cirrhosis patients [11, 33]. This becomes notable because of the rapid depletion of CD8+ cytotoxic T cells specific to decompensated cirrhosis, causing an increased CD4/CD8 ratio when Th1 cells are expanded and activated in both mesenteric lymph nodes and peripheral blood [33, 34]. Therefore, decompensation, in comparison with the compensated state, presents with heightened T‐cell proliferative activity with relative loss of activation and exhaustion marker expression [34]. Despite this inflammatory milieu, the capacity of CD4+ T cells to produce proinflammatory cytokines upon stimulation is strongly diminished in both acute decompensation and acute‐on‐chronic liver failure (ACLF) [35, 36]. This is probably the main mechanism underlying CAID, which predisposes patients with cirrhosis to infections [12, 37].

**FIGURE 1:**
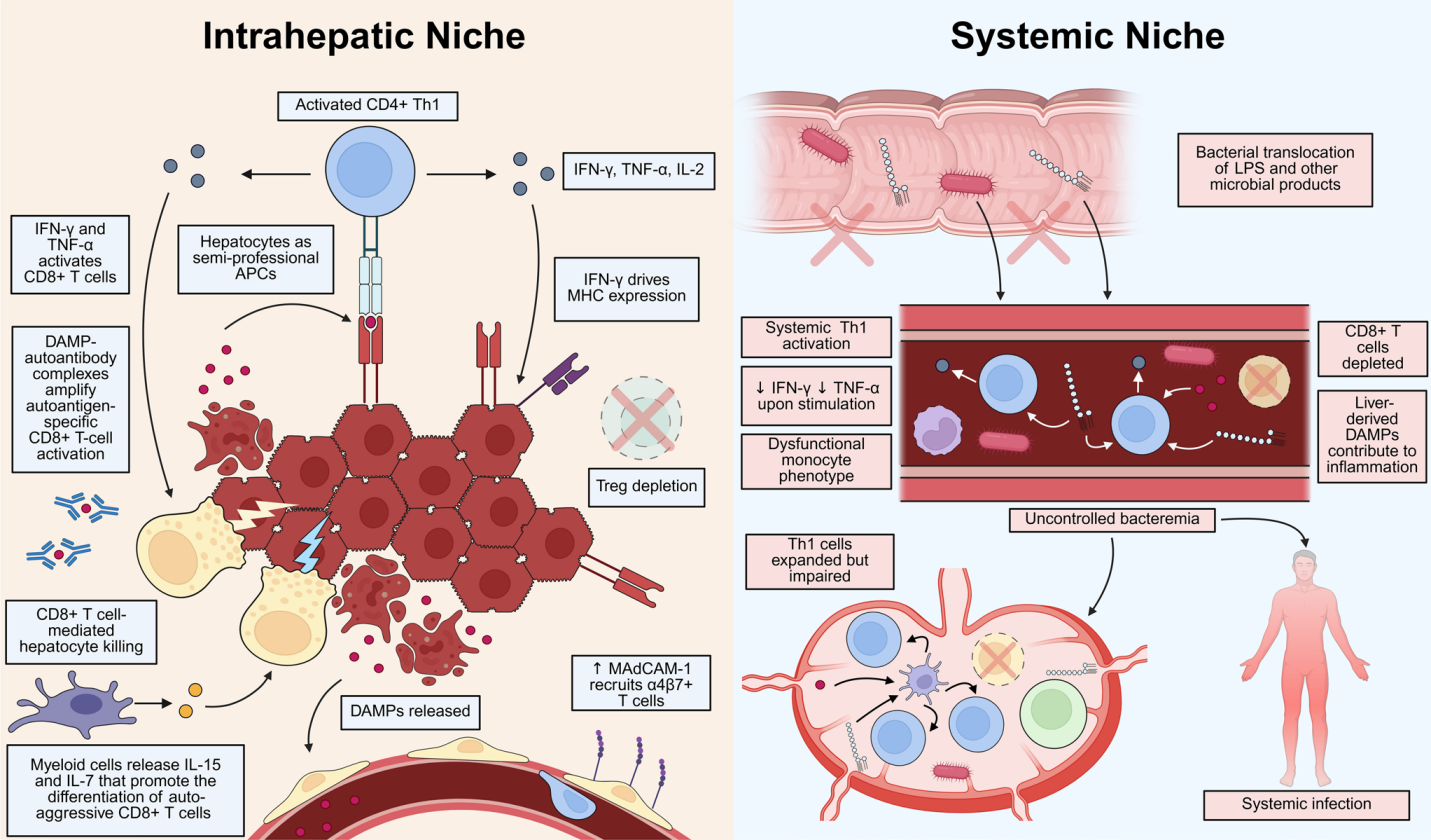
Visual representation of the dual immunological niche in autoimmune‐hepatitis‐related decompensated cirrhosis. In the intrahepatic niche, Th1 cells secrete IFN‐γ, TNF‐α and IL‐2, driving hepatocyte MHC class I/II expression and CD8+ T‐cell‐mediated cytotoxicity. Hepatocyte death releases DAMPs, which form complexes with autoantibodies and amplify T‐cell responses. MHC class II+ hepatocytes present DAMPs/autoantigens directly to CD4+ T cells, creating an amplification loop. LSECs exhibit MAdCAM‐1 upregulation, recruiting α4β7+ T cells. Tregs are deficient, and Kupffer cells/LSECs show reduced efferocytosis. In the systemic niche, bacterial products translocate across an impaired gut barrier, reaching mesenteric lymph nodes and blood. Lymph nodes show Th1 expansion, but CD4+ T cells are functionally paralyzed. Blood contains non‐responsive CD4+ T cells, depleted CD8+ T cells, and circulating DAMPs and PAMPs. Monocytes exhibit impaired phagocytosis. This CAID state leads to recurrent infections (SBP, pneumonia, UTI and bacteremia) as paralyzed immune cells fail to clear pathogens. CAID, cirrhosis‐associated immune dysfunction; DAMP, damage‐associated molecular pattern; IFN‐γ, interferon‐gamma; IL‐2, interleukin‐2; LSEC, liver sinusoidal endothelial cell; MAdCAM‐1, mucosal addressin cell adhesion molecule 1; MHC, major histocompatibility complex; PAMP, pathogen‐associated molecular pattern; SBP, spontaneous bacterial peritonitis; Th1, T helper type 1; TNF‐α, tumour necrosis factor‐alpha; Treg, regulatory T cell; UTI, urinary tract infection.

The convergence of these two Th1‐driven antigenic challenge pathways creates a disconnect between the local and systemic immune compartments (Figure 1). Despite the peripheral immune paralysis and the capacity of cirrhosis liver sinusoidal endothelial cells (LSECs) to suppress T‐cell activity and induce CD8+ T‐cell resistance [38, 39, 40], 89% of patients with AIH‐related decompensated cirrhosis have elevated aminotransferases and modified hepatic activity index scores, indicating ongoing autoimmune activity [41]. Furthermore, intrahepatic T cells in cirrhosis patients display more markers of activation than those in controls do [42].

This apparent paradox could be speculated to derive from diverse mechanisms. Importantly, a recent paper shows that, in AIH, liver‐resident tissue myeloid cells and hepatocytes provide IL‐15 and IL‐7 signals that promote the proliferation, survival and cytotoxic differentiation of auto‐aggressive CD8+ T cells [23]. Liver fibrosis and the consequent upregulation of mucosal addressin cell adhesion molecule (MAdCAM)‐1 have also been shown to induce the enrichment of intrahepatic α4β7+ CD4+ and CD8+ T cells and monocyte recruitment in cirrhosis [43, 44]. Moreover, damage‐associated molecular patterns emerging from hepatocyte death are known drivers of inflammation in the hepatic niche in chronic liver disease [45, 46]. Since AIH hepatocytes aberrantly express HLA class II molecules [47], they can be processed and presented not only by professional antigen‐presenting cells but also by surviving hepatocytes themselves, potentially amplifying autoantigen‐specific T‐cell responses. This could be further enhanced by reduced efferocytosis due to the dysfunctional phenotype of Kupffer cells and LSECs in decompensated cirrhosis [48, 49, 50]. Furthermore, the formation of DAMP–autoantibody complexes may increase the activation of autoantigen‐specific CD8+ T cells [51].

Importantly, this dual immunological niche presents a significant therapeutic dilemma for clinicians. An intuitive approach is to mitigate ongoing liver injury through immunosuppression. Of note, since TNF appears to function as a critical signalling hub, early clinical data have demonstrated that steroid‐free TNF blockade with infliximab can induce biochemical remission in newly diagnosed AIH [23, 52]. A previous study on infliximab as a possible rescue therapy in patients with AIH included seven patients with cirrhosis and reported favourable outcomes, although infectious complications occurred in five of them and data on decompensation was not available [53]. However, there is a prevailing concern that attenuating the hepatic response and systemic inflammation could inadvertently exacerbate the characteristic state of peripheral immune paralysis. This concern is particularly relevant given the role of infection, which serves both as a primary trigger for ACLF and as a leading cause of mortality in decompensated cirrhosis patients [54, 55]. Furthermore, in patients with established cirrhosis secondary to AIH, hepatic inflammation may become ‘burnt‐out’, characterized by reduced necroinflammatory activity and progressive replacement of functional parenchyma by irreversible fibrosis, architectural distortion and hepatocellular loss [56, 57, 58]. At this stage, liver dysfunction predominantly reflects fixed structural damage rather than ongoing immune‐mediated injury, creating substantial uncertainty as to whether the potential benefits of immunosuppression outweigh its risks, including infection, metabolic complications, further clinical decompensation or even LT waitlist dropout.

## 3 The Immunosuppression Minefield in Decompensated Cirrhosis

Since pivotal trials in the 1970s demonstrated the clear survival advantage of corticosteroids over placebo for AIH, immunosuppression has been the standard of care for this condition [2, 59]. Current first‐line therapy aims to induce CBR via predniso(lo)ne in combination with either azathioprine or mycophenolate mofetil, followed by long‐term maintenance with the steroid‐sparing agent alone or with low‐dose predniso(lo)ne [15, 60, 61, 62]. Treatment is recommended for all AIH patients with active disease, including those with advanced fibrosis and/or compensated cirrhosis. However, guidance is less clear for decompensated patients, owing to the differences in study designs, definitions of decompensation and complicated conceptual overlaps [15, 63]. Azathioprine is generally avoided when initiating treatment in patients with decompensated cirrhosis because evidence supporting its safety and efficacy in this setting is limited [15]. Concerns are particularly relevant in patients with severe cytopenias and include the potential for idiosyncratic hepatotoxicity and life‐threatening infections, complications that could jeopardize their eligibility for LT [64, 65]. As such, prednisone is typically the preferred agent for treatment initiation. However, data regarding the optimal corticosteroid regimen in patients with AIH‐related decompensated cirrhosis remain scarce, and no evidence‐based recommendations can currently be made regarding the most appropriate dosing strategy in this setting. A pragmatic maintenance approach has recently been proposed, consisting of the introduction of mycophenolate mofetil or tacrolimus once biochemical and clinical improvement has been achieved [58, 66]. Table 1 summarizes the evidence and practical recommendations derived from the available studies assessing immunosuppression in AIH‐related compensated and decompensated cirrhosis.

**TABLE 1:** Choice and dosing of immunosuppressive agents reported in studies of autoimmune hepatitis‐related cirrhosis. We emphasize that, because no randomized data define an optimal regimen in decompensated AIH cirrhosis, this table is intended as a pragmatic clinical guide rather than a prescriptive dosing algorithm.

Agent	Evidence of use	Evidence on dosing	Evidence on infectious complications	Practical recommendation for decompensated AIH cirrhosis
Prednisone/prednisolone	Universal first‐line induction agent across reported AIH cohorts. Corticosteroids are recommended for active disease, including advanced fibrosis and compensated cirrhosis [15].	Dosing varies widely across studies: median 8 mg/day in one cohort [14], 15–50 mg/day in another [67], 20–30 mg/day according to ascites severity [68], and ≥ 0.5 mg/kg/day in 70% of patients in one series [41]. No consistent dose–response relationship has been established.	Infectious complications are common in decompensated cirrhosis, particularly in patients with gross ascites (12/24 in decompensated AIH with gross ascites; 9/38 in decompensated AIH with no or mild ascites; [68]). In Barbero [14], infection rates were similar between treated and untreated patients (25/72 vs. 18/53), likely reflecting selection bias. In Wang [67], 7/9 deaths in treated patients were attributed to infection. In compensated AIH cirrhosis, infections were reported in 3/32 patients [68]. Among patients who developed invasive candidiasis, 42.9% had cirrhosis at the time of infection (Barrio, 2025).	First‐line therapy in active disease, but in decompensated cirrhosis the safest practical approach is to start with the lowest effective induction dose and taper according to biochemical response rather than fixed weight‐based targets, given the absence of dose‐outcome validation in this subgroup.
Budesonide	Used only in selected non‐cirrhotic AIH populations. It is contraindicated in cirrhosis on mechanistic grounds because portal‐systemic shunting reduces first‐pass hepatic clearance and increases systemic exposure. Furthermore, it is associated with increase portal vein thrombosis risk [15]. This is a blanket cirrhosis contraindication rather than a compensation‐status‐specific one.	Standard AIH dosing is 9 mg/day in eligible non‐cirrhotic patients [15]. No dose‐finding data support use in cirrhosis.	No meaningful cirrhosis‐specific infection data.	Do not use in decompensated AIH cirrhosis [15].
Azathioprine	Used mainly as combination or maintenance therapy. In Barbero [14], 32/72 decompensated AIH cirrhosis patients received azathioprine.	In general AIH protocols, azathioprine is started at 50 mg/day and titrated to 1–2 mg/kg/day [15]. In Barbero [14], dosing was fixed at 50 mg/day. No comparative cirrhosis‐specific dosing data were available.	Cirrhosis‐specific infectious complication data are limited. In decompensated disease, the main practical concerns are cytopenias, intolerance, and difficulty distinguishing drug toxicity from liver‐related abnormalities rather than a clearly quantified infection signal.	Avoid routine initiation at presentation in decompensated disease. It may be considered later, once clinical stabilization is achieved, but benefit–risk balance is uncertain.
Mycophenolate mofetil	Classically used as second‐line therapy/azathioprine intolerant patients in a minority of cirrhotic AIH patients (4/72 in Barbero [14]; part of 11/64 in Wang [67]). In broader AIH cohorts, it is increasingly accepted as an alternative first‐line steroid‐sparing agent, but cirrhosis‐specific data remain scarce [60, 61, 62].	In the AIH literature, 1 g/day was used in Barbero [14], while Wang [67] did not report agent‐specific dosing. General AIH guidance supports 1.5–2 g/day (0.75–1 g twice daily) as the usual dose range [15].	MMF‐related toxicity is generally described as uncommon in AIH overall (with the exception of possible teratogenesis), but infection rates are not reported separately by cirrhosis status [15]. Available AIH studies do not allow a reliable estimate of decompensated‐cirrhosis‐specific infectious risk.	Reasonable steroid‐sparing option once the patient is clinically stable, but evidence in decompensated cirrhosis is indirect and extrapolated from mixed AIH populations.
Tacrolimus	Used mainly in refractory or difficult‐to‐treat AIH. Evidence is limited to small series; in Tannous [66], 36% of patients had cirrhosis and overall remission reached 92%, but outcomes were not reported by fibrosis stage.	EASL [15] suggests 0.1 mg/kg every 12 h with a target trough around 6 ng/mL as a third‐line strategy. This is expert‐panel guidance rather than a cirrhosis‐specific dose‐finding result.	Infection reporting is sparse in AIH series, and available cohorts do not identify infection as the dominant toxicity signal. However, event rates are too small and too inconsistently reported to quantify risk in decompensated cirrhosis.	Not supported by direct evidence in decompensated AIH cirrhosis. If used, apply transplant‐style therapeutic drug monitoring and closely monitor renal function and infection risk.
Rituximab	Very limited evidence in AIH. Published series include a small number of cirrhotic patients, but decompensated‐cirrhosis‐specific data are extremely sparse.	EASL [15] suggests 1000 mg IV at week 0 and week 2, with re‐administration every 6 months if needed. This is expert‐consensus dosing rather than a validated regimen in cirrhosis.	Reported infectious complications in AIH series appear uncommon, but sample sizes are too small for reliable estimates and outcomes are not stratified by compensation status. Risk of infection may be significantly higher in cirrhotic patients	Cannot be recommended on the basis of existing evidence for decompensated AIH cirrhosis; use only in exceptional rescue situations.
Infliximab	Evidence is limited to small observational studies and case series. In Weiler‐Norman [53], 63.6% of patients had baseline cirrhosis, but compensation status was not separated. In the recent multicenter AIH cohort of Efe [52], patients with prior decompensated cirrhosis were excluded.	EASL [15] lists 5 mg/kg at weeks 0, 2, 6 and 10, followed by maintenance every 4–8 weeks. This is panel‐recommended dosing, not cirrhosis‐specific dose validation.	Infection risk is the main concern. In Weiler‐Norman [53], infections were frequent (7/11), with some requiring hospitalization (2/11). In a recent multicenter AIH cohort of 42 patients, 5/42 (12%) had cirrhosis at baseline, patients with prior decompensated cirrhosis were excluded, and 11/42 (26%) developed infections during therapy, including bacterial pneumonia, oral candidiasis, herpes simplex, shingles, urinary tract infection and CMV‐associated colitis [52].	AASLD and EASL caution against use because of weak efficacy evidence and infection risk, particularly in cirrhosis. This caution should be interpreted as applying most strongly to decompensated disease.

Abbreviations: AASLD, American Association for the Study of Liver Diseases; AIH, autoimmune hepatitis; EASL, European Association for the Study of the Liver.

Before initiating immunosuppression, clinicians should recognize that not all patients presenting with clinical decompensation necessarily have established cirrhosis. Acute severe AIH (AS‐AIH), subacute liver failure, AIH‐related acute‐on‐chronic liver failure and severe inflammatory flares with extensive hepatocellular necrosis may present with ascites, encephalopathy, coagulopathy or jaundice that mimic decompensated cirrhosis, yet differ substantially in prognosis and reversibility [69, 70]. When the diagnosis is uncertain and histological findings would meaningfully alter management, liver biopsy interpreted by an expert hepatopathologist may be invaluable for distinguishing established cirrhosis from severe inflammatory injury [63].

Although some studies have attempted to identify predictors of transplant‐free survival (TFS) in decompensated AIH patients treated with immunosuppression (Table 2), the evidence base remains entirely observational—most of which are small single‐center cohorts—rendering these analyses susceptible to selection bias [14, 41, 67, 68]. Indeed, treated patients in these cohorts consistently presented higher baseline albumin and aminotransferases, but were younger and had lower MELD scores, suggesting that clinicians preferentially offer immunosuppression to individuals with more biochemically active disease and preserved synthetic function [15]. Moreover, various studies exclude decompensated cirrhosis patients based on both an attempt to not skew outcomes negatively and an implied inadequacy for treatment [71, 72]. This makes data on this matter very scarce and difficult to interpret appropriately. Such caution is especially important when interpreting the positive findings related to immunosuppression in almost all of these studies, which could be the result of the inclusion of patients with less advanced disease.

**TABLE 2:** Studies assessing predictors of transplant‐free survival in patients with autoimmune hepatitis decompensated cirrhosis.

Study	Author (Year)	Design	Proportion receiving treatment	Treatment details	Significant (*p* < 0.05) differences between treated and non‐treated patients	Outcomes analyzed	Transplant‐free survival predictors
Hepatic Encephalopathy and MELD‐Na Predict Treatment Benefit in Autoimmune Hepatitis‐related Decompensated Cirrhosis (2025)	Arvaniti (2025), Ref [41]	Retrospective multicenter cohort	92.2% (214/232)	All treated patients received prednisolone induction therapy. Most (70%) started ≥ 0.5 mg/kg with guideline‐based tapering. 30% received < 0.5 mg/kg. A second immunosuppressive agent was added in 62% after a median of 1 month. Prednisolone monotherapy was used in 70% during the first month.	Treated patients had higher mHAI grading Treated patients had lower rates of previous variceal bleeding Treated patients had higher AST, ALT, GGT, and bilirubin	Transplant‐free survival Overall survival Adverse events	No/mild overt hepatic encephalopathy (grade ≤ 2) MELD‐Na ≤ 28 MELD‐Na decrease ≥ 11 after 4 weeks of treatment Sufficient biochemical response
Immunosuppressive treatment in autoimmune decompensated cirrhosis, when to say enough: A retrospective analysis (2025)	Barbero (2025), Ref [14]	Retrospective single‐center cohort	57.6% (72/125)	Of the 72 treated patients, 35 received corticosteroid monotherapy, 32 received corticosteroids plus azathioprine, 4 received corticosteroids plus mycophenolate mofetil, and 1 received azathioprine monotherapy. The median prednisone dose was 8 mg/day (IQR 4–20). Azathioprine was administered at 50 mg/day in all patients, and mycophenolate mofetil at 1000 mg/day.	Treated patients were younger Treated patients had lower MELD‐Na Treated patients had lower rates of previous moderate/severe ascites Treated patients had higher AST and sodium	Transplant‐free survival Adverse events	Mild/no ascites MELD‐Na ≤ 22
Presence and type of decompensation affects outcomes in autoimmune hepatitis upon treatment with corticosteroids (2020)	Sharma (2021), Ref [68]	Retrospective single‐center cohort	100% (62/62)	The median daily steroid dose was 25 mg/day (IQR 20–30) in patients with mild or no ascites and 20 mg/day (IQR 20–25) in those with gross ascites. After 6 months, only 15% of decompensated patients remained on prednisolone, most at doses < 10 mg/day.	—	Transplant‐free survival Biochemical remission Adverse events	Mild/no ascites Lower MELD scores
The Management of Autoimmune Hepatitis Patients With Decompensated Cirrhosis: Real‐World Experience and a Comprehensive Review (2017)	Wang (2017), Ref [67]	Retrospective single‐center cohort	78.1% (64/82)	60 of the treated patients started prednisolone induction (15–50 mg/day) tapered to 5–10 mg/day maintenance in a response‐guided manner. Four patients were already on maintenance corticosteroids at decompensation and continued therapy unchanged. Combination therapy with azathioprine or mycophenolate mofetil was used in 11/64 patients.	Treated patients were younger Treated patients had lower 1999 scores Treated patients had higher AST, ALT, Hb, and albumin	Transplant‐free survival Adverse events	Higher total bilirubin reduction 7 days after treatment initiation Higher MELD reduction 7 days after treatment initiation No infections

Abbreviations: AIH, autoimmune hepatitis; ALT, alanine aminotransferase; AST, aspartate aminotransferase; GGT, gamma‐glutamyl transferase; Hb, haemoglobin; LT, liver transplantation; MELD, Model for End‐Stage Liver Disease; MELD‐Na, Model for End‐Stage Liver Disease‐Sodium; mHAI, modified histologic activity index.

Notwithstanding these limitations, the current literature provides useful insights into this matter, suggesting the type and severity of initial decompensating events along with the value and dynamics of prognostic scores (MELD and MELD‐Na) within the first weeks of treatment could predict immunosuppression benefits. A clear trend in predicting TFS is observed in patients with lower MELD scores at baseline. One study suggested a 22‐MELD‐Na cutoff on the basis of a lack of significance for treatment mediating better outcomes in this subgroup, although this cutoff was based on a small sample size (27 patients), which predisposes to type II errors [14, 73]. A better powered study revealed significantly better TFS in treated patients with MELD‐Na ≤ 28, a cutoff previously used to define treatment benefit in patients with acute liver failure due to AIH [41, 74]. This relationship was also consistent in a continuous manner [14, 68]. Importantly, because these analyses are restricted to treated patients, selection bias is unlikely to explain the observed gradient. Rather, these findings likely reflect that patients with greater hepatic reserve are better positioned to recover synthetic function once inflammation is controlled [75].

Notably, this is not unique to AIH. In alcohol‐associated hepatitis, large‐scale cohort studies have defined a therapeutic window for corticosteroids between MELD 25 and 39, with maximal benefit observed in this range and clear futility above MELD 51 [76]. Similarly, in hepatitis C‐related decompensated cirrhosis, antiviral therapy is generally avoided when the MELD score exceeds 25, as the risk of complications outweighs the likelihood of clinical improvement [77].

Another consistent finding has been the deleterious effect of moderate to severe ascites. In AIH, decompensated features of clinically significant portal hypertension—including ascites—are associated with a 15.73‐fold increased hazard of LT or death [78]. This aligns with the broader cirrhosis literature, where nonmild ascites portends poor prognosis irrespective of aetiology and should prompt evaluation for LT, especially when refractory to diuretic therapy [57, 79]. Importantly, ascites is the most common decompensating event in AIH, affecting approximately 89% of patients at the time of decompensation [41]. The convergence of ascites and immunosuppression may create a particularly hazardous scenario. Ascites may partially explain the cycle of bacterial translocation and systemic inflammation, substantially increasing the risk of spontaneous bacterial peritonitis and other infections, which may be amplified by immunosuppression therapy [80, 81, 82]. Given these vulnerabilities, it is biologically plausible that in patients with moderate to severe ascites, the infection risk of treatment outweighs any potential benefit, explaining the observed results.

Similarly, one study noted that overt hepatic encephalopathy (OHE) grade ≥ 3 was the most powerful predictor of poor TFS following immunosuppressive treatment [41]. While OHE is classically recognized as a marker of advanced liver disease and further decompensation state—often carrying a prognostic weight exceeding that of ascites—its role may not be purely associative [57, 83]. OHE can directly lead to complications such as aspiration, falls, and impaired self‐care and is an independent risk factor for *de novo* infections—even after adjustment for several factors—which collides with the need for immunosuppression [84]. Nonetheless, OHE also reflects the severity of underlying liver dysfunction and the presence of precipitating factors (e.g., infection, renal dysfunction and gastrointestinal bleeding), which are themselves associated with poor prognosis [85, 86].

Moreover, high‐grade OHE signals the presence of large spontaneous portosystemic shunts that divert splanchnic and portal blood away from the liver and directly into the systemic circulation, a phenomenon that can account for up to 65% of total hepatic blood flow in advanced cirrhosis patients [87, 88, 89]. Oral immunosuppressants absorbed from the gut normally undergo first‐pass hepatic extraction [90]. However, in the context of extensive portosystemic shunting, a substantial fraction of an administered dose may bypass the liver entirely, reducing intrahepatic drug levels while potentially increasing systemic exposure and toxicity. This pharmacokinetic principle is well established for high‐extraction drugs, such as lidocaine [91]. By contrast, intravenously administered anti‐TNF agents such as infliximab are not subject to intestinal absorption or first‐pass hepatic extraction, so portosystemic shunting is unlikely to alter their bioavailability in the same way it may for orally administered immunosuppressants [92]. Although standard immunosuppressive agents for AIH are thought to have minimal first‐pass hepatic extraction and act more through systemic immune modulation than by requiring high intrahepatic concentrations, this assumption may not hold true for decompensated AIH cirrhosis because of the dual immune niche described earlier [63, 93, 94]. Furthermore, both prednisone and azathioprine require hepatic metabolism to be converted into their active forms [95]. This concern is further compounded by decreased cytochrome P450 enzyme activity in cirrhosis, which can variably affect drug disposition [96]. Nonetheless, evidence is scarce, and this has not been a concern in clinical practice, with the exception of budesonide use, owing to its high reliance on first‐pass hepatic metabolism and higher risk of portal vein thrombosis in cirrhosis [2, 97].

Studies have reported rates of infections of approximately 26%–34.7% for immunosuppressed patients with AIH decompensated with cirrhosis, which is surprisingly equivalent to patients without treatment [14, 41, 68, 98]. However, as previously described, this is probably the result of selection bias, as patients with less severe or more actionable disease are treated over more advanced patients. Furthermore, there is a lack of time‐to‐event and multivariate analyses, which hinders current conclusions. Notably, one study reported that although infection rates were comparable, immunosuppressed patients died nearly 2 times more often (32% versus 16.7%) because of infections [14]. In contrast, the presence of OHE ≥ grade 2 and a higher baseline MELD‐Na score were associated with an increased risk of infection during follow‐up, according to data from the International Autoimmune Hepatitis Group (IAIHG), although only 4% of patients required treatment discontinuation due to infectious complications. These findings underscore that disease severity at presentation, rather than immunosuppression per se, may be the primary driver of infectious risk in this population. Moreover, no significant differences were observed between treated and untreated patients regarding post‐liver transplantation complications. Importantly, higher initial corticosteroid doses were not associated with an increased risk of post‐transplant infections, suggesting that concerns about early intensified immunosuppression may be overstated in carefully selected candidates [41, 58].

Overall, for patients with moderate‐to‐severe ascites and/or OHE ≥grade 2, current evidence and biological plausibility do not support the benefits of immunosuppression over the potential risks and poor prognosis classically associated with these conditions. For this population, immediate referral for LT may be more beneficial, as generally done with AS‐AIH or ACLF [13, 45].

## 4 Assessing Response to Immunosuppression

In addition to their ability to predict response, there is a critical gap in how physicians should assess whether decompensated AIH patients undergoing treatment should continue immunosuppression or be referred for LT. Notably, while biochemical response is associated with better TFS in these patients, CBR assessment can only be performed after at least 6 months of treatment, and studies assessing other AIH‐specific markers of response in decompensated disease patients are scarce [41, 67, 99]. Furthermore, in decompensated cirrhosis, aminotransferases and IgG levels are poor markers of disease activity and/or hepatocellular injury, while autoantibodies are used mostly for diagnostic purposes [100, 101, 102, 103].

Borrowing from a similar dilemma, AS‐AIH researchers have developed several criteria that could be used to assess whether patients respond to corticosteroids. Notably, the SURFASA score—which incorporates the baseline international normalized ratio (INR) and the percentage changes in the INR and bilirubin level over the first 3 days of corticosteroid therapy—is highly predictive of response, with excellent discrimination for LT or death [104, 105]. Nonetheless, the cohort used for the development of the score comprised patients with AS‐AIH irrespective of fibrosis stage, with 21% having cirrhosis at baseline and no data about prior decompensation [104]. Additionally, a MELD‐Na score greater than 26 on day 3 of treatment or a MELD score of 25 on day 7 is associated with high sensitivity (85.7% and 62.5%, respectively) and specificity (78% and 95.2%, respectively) for nonresponse to corticosteroids in this condition [105, 106]. A similar method was used to predict the response to corticosteroids in alcoholic hepatitis patients using the Lille model [107, 108]. As such, early nonresponders can be identified, allowing for quick referral to LT [70].

Although data concerning decompensated AIH are scarce, some dynamic predictors have been assessed. Importantly, decreases in the MELD‐Na score after starting therapy have been associated with improved TFS. Specifically, one study proposed a MELD difference of 1.5 after 7 days of treatment as a good moderator of response, whereas another study reported that a MELD‐Na reduction of ≥ 11 after 4 weeks of treatment had a 100% negative predictive value for death or LT. A total bilirubin reduction > 0.196 mg/dL after 7 days of treatment has also been shown to moderate TFS, although another study did not find this parameter to predict response. A 50% decrease in transaminase levels at 4 weeks of treatment has also been shown to correlate with increased LT‐free survival (92% vs. 64% at 12 months and 88% vs. 53% at 36 months) [41].

Response criteria specific to decompensated AIH are urgently needed. Although the Lille and SURFASA models exclusively assess hepatic function recovery, a logical model for decompensated AIH could incorporate classical short‐term response criteria defined by the International AIH Group [99]. In this patient population, the immediate need for hepatic function restoration is often less critical, whereas the risk of future disease flares requires greater emphasis [9]. However, as mentioned before, laboratory criteria may not be entirely sensible in these patients. Currently, MELD‐Na reductions seem to be the most validated predictor, with biochemical response as a specific supporting factor. In this way, close clinical and biochemical monitoring within the first 4 weeks of treatment is mandatory and failing to achieve a significant reduction in bilirubin and MELD‐Na should prompt immediate LT referral due to the high risk of unfavourable outcomes.

## 5 Liver Transplantation in AIH

Although achieving CBR has been strongly associated with fibrosis regression in AIH patients, LT is currently the only definitive treatment for AIH‐related decompensated cirrhosis—accounting for approximately 3%–5% of transplantations in Europe and the United States [63, 109, 110]. The most common indication for LT in AIH patients is decompensated cirrhosis resulting from chronic hepatocellular failure, although acute (sub)fulminant hepatic failure can also necessitate transplantation [74, 111]. Compared with other chronic liver diseases, hepatocellular carcinoma (HCC) is a less frequent indication of AIH [111]. AIH patients generally have a very favourable prognosis post‐LT, with 10‐year survival estimates of approximately 80% [19, 112].

Despite the excellent outcomes associated with LT in select patients with decompensated AIH cirrhosis, LT is not a definitive cure for AIH. The persistence of host Treg dysfunction means that the autoimmune process can be redirected toward the allograft [113]. Consequently, AIH recurrence affects approximately 21% of transplanted patients, according to recent meta‐analytic data [16]. Unlike primary biliary cholangitis, where recurrence does not appear to compromise graft or patient survival [114], recurrent AIH has been associated with detrimental long‐term outcomes [18, 115]. Managing this risk requires maintaining patients with chronic immunosuppression, which is often more intensive than standard posttransplant protocols, thereby necessitating vigilance for steroid‐related side effects [116, 117].

Nonetheless, AIH has a cumulative incidence of waitlist death or deterioration of approximately 24% at 3 years [118]. Therefore, the decision to transplant patients with decompensated AIH is highly time sensitive and has significant stakes. In the current allocation framework, which relies on the MELD score to rank candidates by objective measures of hepatic dysfunction [119], there is a growing concern in the field that immunosuppressive therapy initiated while on the waiting list may artificially improve laboratory markers of synthetic function, thereby reducing a patient's MELD score and waitlist dropout without proportionally reducing their risk of mortality or infection [13]. However, as discussed earlier, a decreasing MELD score appears to be a valid marker of treatment response in this setting [105, 106]. While evidence for this ‘MELD slippage’ phenomenon is lacking, current data are insufficient to determine whether the observed MELD reduction in treated patients is a sensitive enough biomarker to confidently rule out the possibility that some patients are being systematically disadvantaged.

Even more limited is the evidence on immunosuppression as a bridge to LT. Currently, there are no convincing data suggesting that treating AIH patients awaiting LT improves their likelihood of undergoing transplantation or enhances posttransplant outcomes [109, 115]. Moreover, the immediate posttransplant period is characterized by heightened susceptibility to infection and impaired wound healing, driven by standard post‐LT induction immunosuppression [120, 121]. In particular, AIH patients are even more susceptible to post‐LT infections and graft rejection [122]. Furthermore, evidence is scarce, and heavy pretransplant immunosuppression may reasonably further compromise patients during this vulnerable postoperative phase.

Given these challenges, one could therefore argue that the risks of starting immunosuppression—particularly the potential to delay or complicate transplant eligibility—may outweigh its benefits. For this trade‐off to be justified, there must be evidence that a meaningful subset of patients with AIH‐related decompensated cirrhosis can truly improve on medical therapy alone. This is where the concept of recompensation becomes relevant, offering a framework that could reshape clinical decision‐making in this population.

## 6 The Promise and Peril of Recompensation in AIH

The recent Baveno VII consensus introduced the concept of recompensation, which is defined by at least partial regression of the structural and functional changes of cirrhosis after etiological removal [20]. However, the original consensus was developed primarily from evidence in alcohol‐related liver disease and chronic hepatitis B, in which sustained removal or suppression of the underlying etiological factor can be clearly demonstrated [20, 21, 75]. There is no accepted definition of a cure for AIH. Treatment is generally lifelong, with cessation leading to relapse in 50%–87% of adults and 60%–80% of children [123, 124]. This distinction complicates the application of the original recompensation framework to immune‐mediated liver disease. Consequently, post‐Baveno VII studies have used the CBR as a proxy for etiological suppression, maintaining the other aspects defined by the consensus (e.g., absence of ascites and encephalopathy off therapy) [125, 126].

Various observational studies have attempted to apply the concept of recompensation to AIH, often employing heterogeneous criteria, including pre‐Baveno VII definitions (Table 3). The earliest study defined recompensation simply as the absence of further decompensating events, reporting a 62.5% rate [67]. As definitions evolve to require the absence of prophylactic therapy, this rate decreases to approximately 49% [41]. More recent investigations, which align more closely with the Baveno VII framework by mandating the achievement of CBR alongside clinical stability, have reported substantially lower recompensation rates, ranging from 34.6% to as low as 19% [125, 126, 128]. An exception is the recent Aggarwal et al. [127] paper, which found a 53.6% recompensation rate, despite also using full Baveno VII criteria. However, they did not use CBR to define recompensation and excluded patients who developed decompensation while already on immunosuppression, which probably inflates this proportion [127]. A recent meta‐analysis revealed that approximately 25% of decompensated AIH patients recompensate, with high heterogeneity among studies. This number is lower than etiologies where the concept of an etiological cure can be applied, such as alcohol‐related (33%), hepatitis B infection (50%) and hepatitis C infection (42%) [129]. Crucially, patients with AIH who achieve recompensation have been shown to experience improved long‐term outcomes, including significantly higher transplant‐free survival and a lower incidence of subsequent decompensating events [9, 41, 126, 127, 128].

**TABLE 3:** Published reports assessing recompensation in autoimmune hepatitis decompensated cirrhosis.

Study	Author (Year)	Recompensation definition	Percentage of recompensation among treated patients	Significant (*p* < 0.05) differences between recompensated and non‐recompensated patients	Predictors of recompensation in multivariate analyses	Post‐recompensation outcomes
Recompensation and Further Decompensation After Index Decompensation in Patients with Autoimmune Hepatitis: A Real‐World Study (2026)	Aggarwal (2026), Ref [127]	Resolution of ascites (off diuretics), OHE (off lactulose/rifaximin), and no recurrent variceal bleed for 12 months, together with improvement in liver function to Child‐Pugh Class A. (Biochemical response was analyzed as a separate predictor, not folded into the definition itself.)	53.6% (60/112)	Higher baseline ALT Lower INR Lower LSM Lower MELD‐Na More likely to have AST/ALT normalization at 6 months	Younger age (sHR 0.98, 95% CI 0.95–1.00) Lower Child‐Turcotte Pugh score (sHR 0.81, 95% CI 0.71–0.93) AST/ALT normalization at 6 months (sHR 1.75, 95% CI 1.02–2.88)	9/60 (15%) developed further decompensation (1‐year incidence 3.4%, 2‐year 8.5%) Recompensation associated with reduced mortality (HR 0.22, 95% CI 0.05–0.90) vs. non‐recompensated
Predictors of recompensation in immunosuppressive therapy for biopsy‐proven autoimmune hepatitis patients with decompensated cirrhosis (2026)	Zhang (2026), Ref [128]	CBR (ALT/AST/IgG normalization) or histological remission at 6 months + resolution of decompensation ≥ 12 months + stable improvement in albumin/bilirubin/INR	34.6% (28/81)	Higher BMI Higher ALT/AST Higher albumin Lower diabetes prevalence Lower baseline IL‐17A, TNF‐α, IFN‐γ	BMI (HR 1.161, 95% CI 1.022–1.326) Diabetes as negative predictor (HR 0.582, 95% CI 0.294–0.896) ALT (HR 1.168) ALB (HR 1.388, 95% CI 1.195–1.635) CBR at 6 months (HR 1.895, 95% CI 1.154–2.312)	Only 1/28 died (vs. 10/53 non‐recompensated, log‐rank *p* = 0.044 for survival) 2 patients redeveloped decompensation after recompensation
Extending the Baveno VII definition of recompensation to autoimmune hepatitis: Considerations regarding treatment timing and response criteria (2026)	Hofer (2026), Ref [125]	(1) Sustained absence of decompensation events for ≥ 12 months following cessation of diuretics and prophylactic therapies, accompanied by restoration of Child‐Pugh A liver function; and (2) CBR with normalization of aminotransferase and IgG levels under immunosuppression	19% (4/21)	—	—	All 4 recompensated patients remained alive and not transplanted at last follow‐up (5.2–16.9 years). In contrast, among nonrecompensated patients, 6/17 died and 4/17 required transplantation. Redecompensation after recompensation was not observed.
Recompensation in patients with autoimmune hepatitis‐related decompensated cirrhosis following immunosuppressive therapy (2025)	Chen (2025), Ref [126]	(1) Sustained absence of decompensation events for ≥ 12 months following cessation of diuretics and prophylactic therapies, accompanied by restoration of Child‐Pugh A liver function; and (2) CBR with normalization of aminotransferase and IgG levels under immunosuppression	30.9% (68/220)	Lower IgG levels at baseline Higher ALT levels at baseline Higher platelet counts at baseline Ascites as the first decompensating event Variceal bleeding not being the first decompensating event Ascites being the only decompensating event during follow‐up Variceal bleeding and encephalopathy not occurring during follow‐up Longer immunosuppressant treatment duration	Lower IgG at baseline (HR 1.09, 95% CI 1.04–1.12) Higher platelet count at baseline (HR 1.00, 95% CI 1.00–1.01) Higher total bilirubin at baseline (HR 1.04, 95% CI 1.00–1.08) Ascites as the sole decompensating event (HR 14.40, 95% CI 4.17–49.64)	Lower risk of liver transplantation or death than those not recompensated (HR 0.07, 95% CI 0.01–0.50) Survival comparable to those of compensated patients 5.9% experienced further decompensation after recompensation
Hepatic Encephalopathy and MELD‐Na Predict Treatment Benefit in Autoimmune Hepatitis‐related Decompensated Cirrhosis (2025)	Arvaniti (2025), Ref [41]	Absence of ascites after diuretic discontinuation and/or (depending on the initial presence) the absence of OHE after resolution of the initial event once prophylactic treatment was withdrawn, and the absence of recurrent variceal bleeding (for at least 12 months).	49% (105/224)	Higher AST levels at baseline Higher ALT levels at baseline Higher total bilirubin levels at baseline	—	Lower risk of liver transplantation or death than those not recompensated (99% vs. 49% at 12 months and 97% vs. 37% at 36 months)
The Management of Autoimmune Hepatitis Patients With Decompensated Cirrhosis: Real‐World Experience and a Comprehensive Review (2017)	Wang (2017), Ref [67]	Absence of decompensation events after therapy, with or without prophylaxis.	62.5% (40/64)	Male sex Higher ALT levels at baseline Higher AST levels at baseline Higher GGT levels at baseline Higher white blood cell count at baseline Higher Hb at baseline Splenomegaly at baseline No presence of/less severe ascites Higher prednisolone doses	—	—

Abbreviations: AIH, autoimmune hepatitis; ALT, alanine aminotransferase; AST, aspartate aminotransferase; CBR, complete biochemical response; CI, confidence interval; GGT, gamma‐glutamyl transferase; Hb, haemoglobin; HR, hazard ratio; IgG, immunoglobulin G; LT, liver transplantation; OHE, overt hepatic encephalopathy.

Nonetheless, owing to the extended nature of this definition, whether these patients have the same favourable outcomes as classic Baveno VII‐recompensated patients remains uncertain. The beneficial outcomes in patients with AIH that have yet to be extensively validated include a lower incidence of HCC, decreased hyperdynamic circulation, normalization of systemic inflammatory markers and fibrosis regression beyond stabilization [130, 131, 132]. Although CBR in AIH has been shown to induce fibrosis regression and decrease portal hypertension‐related events, which could be reasonably inferred for patients under these extended recompensation criteria, the peculiar immune phenotype of AIH‐related decompensated cirrhosis raises the question of whether such outcomes are still possible at this stage [10, 110, 133]. Furthermore, the use of the CBR to define recompensation has been questioned because elevated immunoglobulins are fairly common in patients with compensated cirrhosis owing to the previously described inflammatory milieu [125, 134]. The extent to which reported recompensation rates may vary according to the biochemical proxy used is well illustrated by a recent large real‐world cohort that defined biochemical improvement based on AST/ALT normalization rather than full CBR and reported a recompensation rate of 53.6%, considerably higher than in CBR‐anchored studies [127]. Moreover, recompensated AIH patients have been shown to have significantly lower baseline levels of pro‐inflammatory cytokines (IL‐17A, TNF‐α and IFN‐γ) than those remaining decompensated, suggesting that a less active baseline inflammatory milieu, not captured by CBR or IgG alone, may be a precondition for achieving a durable recompensated state [128].

In this sense, although recent AIH guidelines are shifting toward decreasing biopsy indications [15], some defend that true recompensation criteria, such as the remission criterion from the International AIH Group (modified hepatitis activity index ≤ 3), would need to follow histology [99, 125]. It has been demonstrated that a considerable proportion of patients with AIH cirrhosis do present histological disease activity despite normal ALT and/or IgG levels, which has been associated with significantly worse outcomes [135, 136, 137]. This is significantly different from the effects of other etiological suppression proxies. In hepatitis C infection, a sustained virological response is strongly correlated with fibrosis regression and histological improvement [138, 139]. Similarly, alcohol abstinence generally leads to histological quiescence [140, 141, 142]. Although liver event analyses of patients with recompensated AIH are lacking, 57% of patients with primary biliary cholangitis‐related decompensated cirrhosis, an autoimmune liver disease with similarly difficult recompensation definitions, eventually presented with liver‐related complications [143].

Although these observations support a potential role for histological assessment beyond diagnosis, we do not advocate routine repeat liver biopsy in all patients with decompensated AIH cirrhosis. Rather, repeat biopsy should be individualized and reserved for situations in which histological information is expected to change management, such as uncertainty regarding residual inflammatory activity despite biochemical remission, given that a substantial proportion of cirrhotic AIH patients with normal ALT and IgG levels continue to exhibit clinically relevant histological activity, discordance between biochemical and clinical or elastography findings, or consideration of substantial immunosuppression tapering [137].

Taken together, these observations suggest that recompensation in AIH patients—using the CBR as a proxy for etiological control—may represent a distinct and more fragile entity than the classic Baveno VII recompensation observed in alcohol‐ or virus‐related cirrhosis patients. Although inconvenient and still imperfect, a histology‐based definition would align more closely with the original consensus intent and might identify a subset of patients in whom immunosuppression could be safely tapered to low‐dose glucocorticoids or azathioprine monotherapy without constant risk of relapse [136]. However, the clear survival benefit observed in patients who achieve this state makes it a worthwhile goal. Thus, the central challenge in clinical practice becomes the timely identification of patients who are candidates for a trial of immunosuppression versus those for whom immediate transplant referral is the safer path [13].

## 7 To Suppress or to Transplant?

**FIGURE 2:**
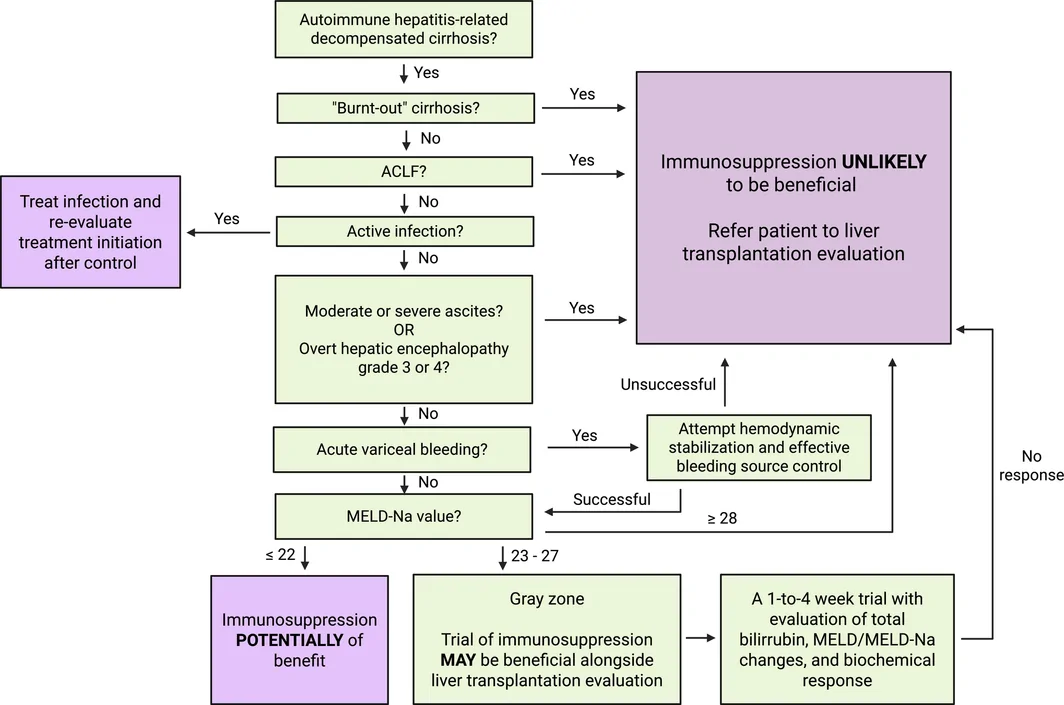
Summary of current evidence for the pretreatment selection of autoimmune hepatitis patients with decompensated cirrhosis with the highest odds of responding to immunosuppression. Patients with active infection, acute‐on‐chronic liver failure (ACLF) or multiorgan dysfunction are considered poor candidates for immunosuppression and should undergo urgent liver transplantation (LT) evaluation. Among clinically stable patients, those with moderate‐to‐severe ascites and/or overt hepatic encephalopathy (OHE) grade ≥ 3 are at high risk of treatment failure and infectious complications and should generally be prioritized for transplant assessment. Patients with mild ascites, low‐grade OHE, or stabilized after acute variceal bleeding without active infection may be considered for a short, protocolized trial of immunosuppressive therapy. Baseline MELD‐Na may be used as a prognostic modifier. Values ≤ 22 appear to be associated with a greater likelihood of transplant‐free survival, whereas MELD‐Na ≥ 28 is consistently associated with poor outcomes despite therapy. Patients within the intermediate ‘grey zone’ (MELD‐Na 23–27) require individualized decision‐making and close monitoring. All thresholds are derived from retrospective observational cohorts and should be interpreted as hypothesis‐generating rather than definitive treatment cutoffs.

From a clinical standpoint, a patient with decompensated AIH cirrhosis can progress toward one of three main distinct endpoints: recompensation (either stable or with future decompensation), liver transplantation or death before any of the two. The central challenge and focus of this review is to accurately allocate patients to the pathway most likely to yield a favourable outcome. The evidence supports an individualized, risk‐stratified strategy. On the basis of the available data and biological plausibility, we propose a framework built on a pretreatment assessment to identify patients with a realistic chance of benefiting from immunosuppression and a time‐limited trial with early, objective stop rules to permit timely referral for transplantation in nonresponders (Figure 2).

The data consistently point to a therapeutic window for immunosuppression in decompensated AIH patients [14, 41, 126]. Since there is no evidence that this course of action serves as an effective bridge‐to‐transplantation, the main goal is recompensation [21, 109, 115]. However, it is equally important to define who lies outside this window. Patients with active infection and ACLF should not be considered for a trial of immunosuppression, as the risk of exacerbating systemic inflammation or precipitating fatal sepsis is usually prohibitive [70, 144].

A reasonable next step is to stratify patients on the basis of the type/severity of cirrhosis decompensation. As previously discussed, patients with moderate–severe ascites and those with OHE grade ≥ 3 have a poor response to immunosuppressants [14, 41, 68]. Considering the high risk in this population, quick referral to LT may be more beneficial in this scenario. Although there have been reports that patients with previous variceal bleeding episodes are less likely to receive pre‐LT treatment and to achieve recompensation upon receiving [41, 125], data on patients with this specific decompensating event are scarce. Even though acute variceal bleeding is a marker of clinically significant portal hypertension and advanced disease, in some patients it may be the sole decompensating event, with otherwise relatively preserved liver function [145, 146]. In such cases, portal hypertension may be partly driven by active inflammatory injury and could plausibly improve with effective immunosuppression, although direct evidence remains limited. Accordingly, after hemodynamic stabilization and in the absence of infection, multiorgan failure or other contraindications, these patients should not be excluded a priori from an immunosuppressive trial. Therefore, the patients most clearly suitable for an immunosuppression trial appear to be those with mild ascites or encephalopathy, while selected patients with isolated variceal bleeding may also be considered on a case‐by‐case basis.

As a final moderator, current data support the use of biomarker‐based stratification, with the MELD score and its variations being the most cited. Nonetheless, data are still scarce. Notwithstanding this uncertainty, evidence shows that patients with MELD‐Na ≥ 28 are unlikely to benefit from this intervention and that patients with values ≤ 22 have reasonable chances of responding [14, 41, 67]. In addition to MELD‐Na possibly reflecting patients' immune compromise and susceptibility to infections [147, 148], this may indicate that some hepatic function is still necessary for patients to achieve recompensation, with further cirrhosis stages being associated with a currently irreversible stage that is better suited for LT [21, 75]. However, the data are less clear in patients with MELD‐Na scores between 23 and 27. For this ‘grey zone’, a binary decision is insufficient. Instead, an individualized expert assessment based on a short‐term, dynamic trial of therapy with strict stop rules may be the best step.

Baseline aminotransferase levels have also emerged as potential predictors of treatment response (Table 3). Several observational studies have reported higher AST and ALT levels among patients who subsequently achieved recompensation or favourable outcomes, possibly reflecting a greater proportion of reversible immune‐mediated injury relative to irreversible architectural damage [41, 67, 126]. However, this association has been inconsistent, has not been independently associated with TFS, and has not remained an independent predictor of recompensation after multivariable adjustment. Therefore, although elevated aminotransferases may provide supportive evidence of ongoing inflammatory activity, they should not currently be used in isolation to select candidates for immunosuppressive therapy. Likewise, other factors associated with disease progression in AIH, including age, sex, and ethnicity, have limited evidence supporting their prognostic value in the setting of decompensated AIH cirrhosis [67, 149, 150]. Accordingly, risk stratification should remain focused on clinical severity and hepatic reserve until prospective studies identify and validate additional prognostic markers.

**FIGURE 3:**
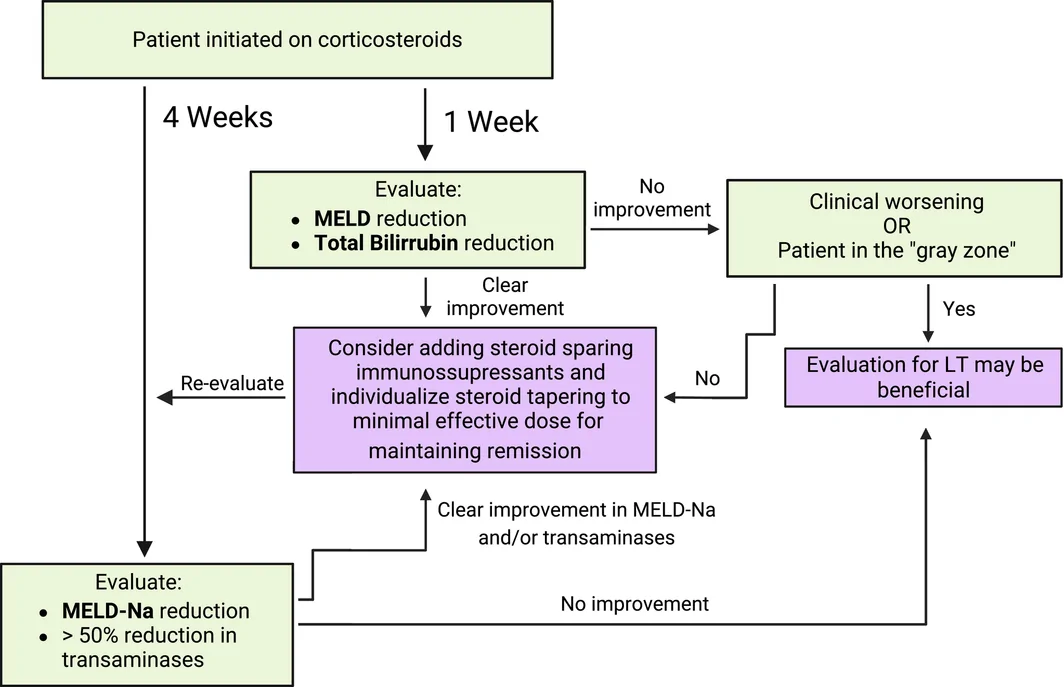
Summary of current evidence from posttreatment initiation assessment of the response of autoimmune hepatitis patients with decompensated cirrhosis receiving immunosuppressive therapies. Two key evaluation time points are highlighted: Week 1 and week 4 after treatment initiation. At week 1, early biochemical trends, including changes in total bilirubin and the MELD‐Na score, may provide preliminary insight into the trajectory. Small cohort data suggest that modest reductions in bilirubin and the MELD‐Na score may be associated with improved transplant‐free survival. However, these early thresholds remain insufficiently validated and should primarily inform closer monitoring or earlier transplant referrals in clinically unstable patients. At week 4, a more robust assessment is recommended. A decrease in MELD‐Na (with each 1‐point reduction associated with a relative reduction in the hazard of transplantation or death in retrospective studies) and evidence of a biochemical response (≥ 50% reduction in aminotransferases from baseline) are associated with improved outcomes. Failure to demonstrate improvement in hepatic function or biochemical activity by week 4 should prompt expedited LT evaluation to avoid delays in definitive management. All the proposed response criteria are derived from observational data and lack prospective validation. Therefore, this proposed algorithm emphasizes early identification of nonresponders and predefined stop rules rather than prolonged empiric continuation of immunosuppression in high‐risk individuals.

The early identification of future nonresponders allows the timely discontinuation of futile treatments. Two key time points for response assessment in patients undergoing a trial of immunosuppressants have been cited in the literature: week one and week four post‐initiation (Figure 3). The initial assessment at 1 week should focus on dynamic changes in total bilirubin and MELD‐Na from baseline. While a MELD reduction of 1.5 points and a total bilirubin variation > 0.196 mg/dL have been associated with 78.6% and 92.9% sensitivity for treatment response, respectively, these thresholds were derived from a small sample size and remain insufficiently validated [67]. As such, the one‐week evaluation may be most valuable in specific contexts, such as for patients in the prognostic ‘grey zone’ or those with deteriorating clinical status, where earlier insight is needed.

A more robust evaluation can be performed at week 4 on the basis of data from a multicenter cohort. At this time point, both a MELD‐Na reduction (with each 1‐point reduction decreasing the hazard of LT or death by 11%) and a biochemical response (transaminase reduction > 50%) should be evaluated [41, 67, 99]. Although the original study emphasized a MELD‐Na reduction of ≥ 11 points because of its 100% negative predictive value, a reduction of ≥ 2 points would already correspond to an estimated > 20% decrease in the hazard of LT or death, which is a common threshold used to define a clinically meaningful effect [151]. Patients who fail to meet these response criteria would probably benefit from undergoing prompt evaluation for LT to avoid delays in definitive management.

## 8 Unanswered Questions and Future Directions

The current evidence base for AIH‐related decompensated cirrhosis, while informative, is largely retrospective and heterogeneous, leaving several critical questions unanswered. Addressing these gaps will require a multifaceted research agenda.

First, the fundamental pathophysiology of the dual immune niche in decompensated AIH cirrhosis remains poorly characterized. While we hypothesize that there is a disconnect between intrahepatic inflammation and systemic immune paralysis, the precise cellular and molecular mechanisms driving this phenomenon are unknown. Future studies should leverage advanced techniques, such as single‐cell RNA sequencing and multiplex immunohistochemistry, on paired liver and peripheral blood samples from well‐phenotyped patients across the spectrum of decompensation [152, 153]. Key questions include the following: What is the functional phenotype of intrahepatic T cells in this population? Does successful immunosuppression restore Treg function or merely suppress effector responses [154, 155]?

Second, the field suffers from a lack of validated, dynamic biomarkers to guide therapy. The proposed cutoffs for early changes in bilirubin and the MELD‐Na score are derived from few studies and lack external validation [41, 67]. A critical priority is the establishment of large, prospective, multicenter cohorts of patients with decompensated AIH initiating immunosuppression. Such cohorts would allow for the validation and refinement of existing early response criteria. Furthermore, the development of novel biomarker panels that might include circulating markers of inflammation (e.g., cytokines) or bacterial translocation (e.g., LPS‐binding protein) may improve the prediction of response and infection risk [156, 157, 158]. Exploration of pharmacokinetic–pharmacodynamic relationships to understand how portosystemic shunting and impaired hepatic metabolism affect drug levels and clinical outcomes, potentially guiding dose adjustments, is also needed [159, 160].

Third, a major conceptual hurdle is the definition of recompensation in AIH patients. The current adaptation of the Baveno VII criteria, which uses the CBR as a proxy for etiological control, is likely insufficient. Persistent histological activity in the face of CBR [135, 136] and the high risk of relapse after treatment cessation [123] suggest that ‘recompensation’ may be more brittle in AIH patients than in those with other liver diseases. Research must determine whether a histologically defined remission (e.g., mHAI ≤ 3) should be a required component of recompensation criteria. Long‐term follow‐up studies comparing the outcomes of patients who meet clinical versus histological recompensation criteria are urgently needed to determine which definition best predicts freedom from future decompensation, HCC and liver‐related death.

Fourth, the optimal design of immunosuppressive regimens for this specific population is unknown [161]. Are lower starting doses of corticosteroids safer [13, 162]? Does the addition of a steroid‐sparing agent from the outset improve outcomes or simply increase toxicity [163]? Comparative effectiveness research, embedded within prospective registries, could begin to answer these pragmatic questions. Furthermore, the role of emerging therapies, such as biologics targeting specific cytokines, warrants exploration, particularly for patients who fail standard therapy but are not yet transplant candidates [164].

Finally, and most importantly, the central question—to suppress or transplant?—can only be definitively answered by further prospective data or randomized controlled trials. While challenging, such a trial is not impossible. One potential design could randomize patients within the ‘therapeutic window’ (e.g., MELD‐Na 23‐27, no severe ascites or OHE) to either a standardized immunosuppressive regimen with strict stop rules (as proposed in Figure 3) or to immediate LT listing with best supportive care. Given the rarity of the condition, such an effort would require a large, international consortium. However, without this approach, clinicians will remain dependent on low‐quality evidence, and patients will continue to face decisions with life‐altering consequences on the basis of uncertainty.

## Author Contributions

**Valbert Oliveira Costa Filho:** conceptualization, methodology, data curation, writing – original draft, formal analysis, investigation. **Guilherme Grossi Lopes Cançado:** conceptualization, supervision, validation, writing – review and editing, project administration, methodology, data curation. **Pedro Robson Costa Passos:** conceptualization, methodology, investigation, writing – review and editing, writing – original draft, formal analysis, data curation.

## Funding

The authors have nothing to report.

## Conflicts of Interest

The authors declare no conflicts of interest.

## Data Availability

The data that support the findings of this study are available from the corresponding author upon reasonable request.
